# Frailty and Inflammatory Bowel Disease: A Scoping Review of Current Evidence

**DOI:** 10.3390/jcm12020533

**Published:** 2023-01-09

**Authors:** Anne Fons, Kees Kalisvaart, Jeroen Maljaars

**Affiliations:** 1 Department of Gastroenterology and Hepatology, Leiden University Medical Centre, 2333 ZA Leiden, The Netherlands; 2 Department of Geriatric Medicine, Spaarne Gasthuis, 2035 RC Haarlem, The Netherlands

**Keywords:** inflammatory bowel disease, Crohn’s disease, ulcerative colitis, frailty, elderly, geriatric assessment

## Abstract

Frailty is increasingly recognized as an important concept in patients with Inflammatory Bowel Disease (IBD). The aim of this scoping review is to summarize the current literature on frailty in IBD. We will discuss the definition of frailty, frailty assessment methods, the prevalence of frailty, risk factors for frailty and the prognostic value of frailty in IBD. A scoping literature search was performed using the PubMed database. Frailty prevalence varied from 6% to 53.9%, depending on the population and frailty assessment method. Frailty was associated with a range of adverse outcomes, including an increased risk for all-cause hospitalization and readmission, mortality in non-surgical setting, IBD-related hospitalization and readmission. Therefore, frailty assessment should become integrated as part of routine clinical care for older patients with IBD.

## 1. Introduction

The prevalence and incidence of inflammatory bowel disease (IBD) is increasing among all age groups, but especially in older people [1]. Currently, the prevalence of IBD in the population of 60 years and older is estimated to increase incrementally annually by 5.2% [2]. Older patients with IBD are generally considered a heterogenous population who are frequently affected by comorbid conditions, polypharmacy, malnutrition and sarcopenia [3]. Frailty is a concept that is increasingly used to address this heterogeneity in health status of people. Frailty represents a dynamic process of decline in functioning across multiple physiological systems, accompanied by an increased vulnerability to adverse health outcomes [4]. Increasing evidence demonstrates that this also applies for IBD, as frailty is associated with a wide range of adverse outcomes in patients with IBD [5].

Currently, there is no consensus on a standardized definition or measure of frailty. In fact, a variety of definitions and methods to asses frailty are used in the literature [6,7]. Some frailty assessment methods rely on data from medical records, while others measure components of frailty directly in patients (such as handgrip strength and walking speed, components of Fried’s Frailty criteria) [7,8]. The lack of a uniform definition and assessment method can impede the implementation of frailty in clinical care for patients with IBD and in guidelines.

The aim of this review is to summarize current literature on frailty in IBD. First, we will discuss what frailty is, the frailty assessment methods and review the prevalence of frailty in patients with IBD. Second, we will discuss risk factors for frailty in patients with IBD. We will outline the association between frailty and generic outcomes (e.g., mortality) and IBD-specific clinical outcomes (e.g., surgery, hospitalizations).

## 2. Materials and Methods

**Figure 1:**
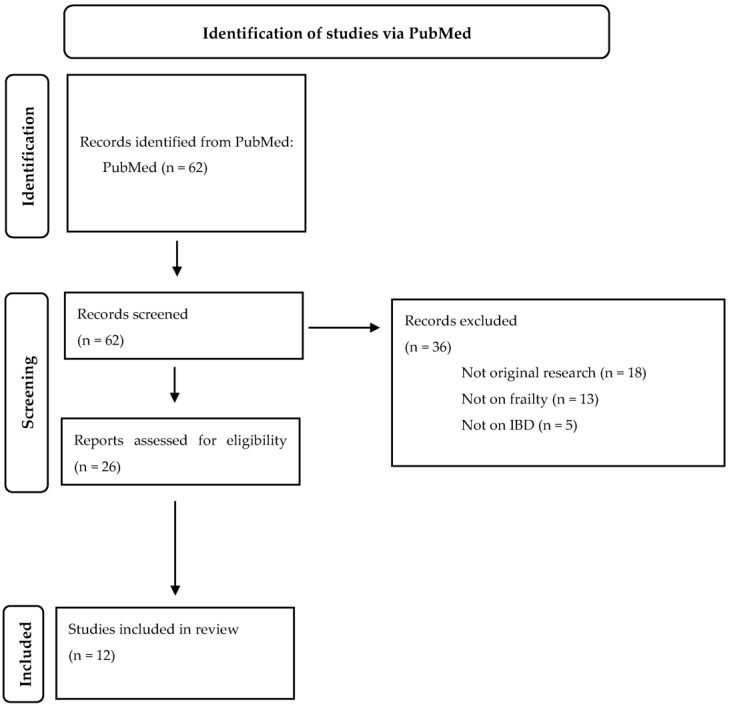
Flowchart.

The study design of a scoping review was considered most suitable to explore multiple, emerging key concepts for frailty in IBD [9]. A literature search was conducted on the 10th of October 2022 using the electronic database PubMed to identify relevant English language articles. In addition, reference lists of the identified articles were screened for additional studies. The Preferred Reporting Items for Systematic Reviews and Meta-analysis (PRISMA) extension for scoping reviews was followed. The search strategy was developed with a specialized research librarian. The following search strategy was used: (((“Frail Elderly” [Mesh]) OR (Frail Elderly)) OR ((((“Frailty” [Mesh]) OR (frail)) OR (frailty)) AND ((((old) OR (older)) OR (elderly)) OR (“Aged” [Mesh])))) AND (((((((“Inflammatory Bowel Diseases” [Mesh]) OR (inflammatory bowel disease)) OR (IBD [tiab])) OR (Crohn’s disease)) OR (ulcerative colitis)) OR (“Colitis, Ulcerative” [Mesh])) OR (“Crohn Disease” [Mesh]))**.** This initial search identified 62 articles, of which 26 articles were read. Original articles were considered eligible if 1) frailty was assessed in patients with IBD and 2) frailty was related to clinical outcomes. Additionally, we included one submitted manuscript provided by one of the co-authors, as we considered the outcomes of this manuscript relevant to discuss in this literature review. Finally, 12 articles were included in this review (Figure 1).

## 3. Results

### 3.1. What Is Frailty?

Frailty is a condition that is characterized by a decline in multiple physiologic systems resulting in a state of increased vulnerability to adverse health outcomes [10]. In literature, there is no consensus on a standardized operational definition of frailty [11]. Definitions differ based on the construct that is chosen [12]. Two definitions are predominantly used in the literature [6]. ‘’Phenotypic frailty’’ or physical frailty, proposed by Fried et al. [4], is defined as “a medical syndrome with multiple causes and contributors that is characterized by diminished strength, endurance, and reduced physiologic function that increases an individual’s vulnerability for developing increased dependency and/or death.’’ [13,14]. Physical frailty is identified when at least three out of the following five key clinical symptoms are present: involuntary weight loss, weakness, fatigue, low levels of physical activity and reduced walking speed [4,6]. Physical frailty is considered preventable and reversible by intervention(s), as will be discussed later in this review.

The accumulation deficits model considers frailty as a multidimensional state of risk which results from acquired, accumulated deficits across multiple domains [15]. This model assumes that the number rather than the nature of health problems leads to an increased state of risk or frailty [6]. Both models are predictive for adverse outcomes in a diverse pallet of medical conditions, however they probably identify significantly different patient populations as a consequence of different theoretical frameworks and frailty measures [14,16]. Several frailty assessment methods have been developed based on these two models [17]. Both models are alternately used in the articles referred to in this literature review. Therefore, if not specified otherwise, when we refer to frailty we imply the global concept of a multidimensional decline associated with increased vulnerability to adverse health outcomes.

When examining these two models in context of IBD, both are relevant and can be applied. For example, multiple components of the frailty phenotype such as fatigue, weight loss and weakness are commonly seen in IBD [4,5]. On the other hand, extra-intestinal disease manifestations, but also other comorbid conditions such as cardiovascular disease and neuropsychological disorders, are prevalent in patients with IBD and contribute to the ‘’accumulation of deficits’’ [18].

Although frailty is an ageing-related syndrome and its prevalence increases with age, it does not only manifest in older patients [19]. Frailty could be considered as a proxy for accelerated biological ageing, irrespective of chronological age [20]. This can be explained by the significant heterogeneity that exists in the rate of biological ageing between patients [21]. Several processes, including low-grade inflammation, are linked to this acceleration of biological ageing [22]. As a result, the biological age can exceed the chronological age, thereby increasing the risk for ageing-related diseases in chronologically younger patients. This effect of biological ageing might also be observed in patients with IBD, where the onset of several geriatric syndromes, including osteoporosis, are seen in relatively young patients [5,23].

Frailty shares a significant overlap with several conditions, including sarcopenia [24,25]. Sarcopenia can occur as a distinct clinical entity, but can also be causally related to frailty [14]. We will briefly outline the characteristics of sarcopenia and its relationship with frailty. Sarcopenia is defined as “a progressive and generalized skeletal muscle disorder that is associated with increased likelihood of adverse outcomes including falls, fractures, physical disability and mortality” [26]. A diagnosis of sarcopenia is confirmed by the presence of low muscle strength and low muscle mass or quality [26]. Several assessment methods exist to measure sarcopenia, and the choice of instrument depends on the purpose, patient population and setting [26]. Methods can roughly be divided into techniques that measure muscle mass (e.g., Computed Tomography, Magnetic Resonance Imaging and Bioelectrical Impedance Assessment) and techniques that measure muscle function (e.g., handgrip strength, walking speed) [26]. The etiology of sarcopenia can be ageing-related, but also secondary to other conditions, for example to malignancy [26]. Sarcopenia is also prevalent in patients with IBD—a recent systematic review found a prevalence of 42%—and is associated with adverse outcomes such as adverse events following surgery [27]. Although sarcopenia and frailty share a significant overlap, they are two distinct concepts [28]. Low physical function is the key characteristic that is shared by sarcopenia and frailty. However, frailty represents a broader, more multifaceted concept than sarcopenia [28].

### 3.2. How to Assess Frailty?

Multiple frailty assessment methods have been developed and validated to identify frailty in both clinical and research settings [29,30]. The Comprehensive Geriatric Assessment (CGA) is considered as the ‘’gold standard’’ to assess the presence of frailty [31]. The CGA is a multidisciplinary, diagnostic and treatment process that systematically assesses four geriatric domains: the somatic, mental, physical and social domain [32]. The CGA comprises both the detection of deficits in geriatric domains and subsequently the initiation of tailored intervention strategies [31]. However, the performance of a CGA in all older patients is not time or cost efficient. Therefore, frailty screening can be performed to identify patients at an increased risk for frailty, requiring referral to a geriatrician for a CGA [33]. Frailty screening methods can be categorized into two types: direct and indirect. Direct screening methods include the performance of screening questionnaires or tests directly on a patient. Commonly used screenings indicators include the FRAIL scale, Clinical Frailty Scale (CFS), Vulnerable Elders Survey 13 (VES-13) and the Geriatric-8 (G8) [29]. The CFS has gained considerable attention during the COVID-19 pandemic, when screening for frailty had to be feasible and simple to perform [10]. The CFS ranges from 1 (very fit) to 9 (terminally ill) [34]. One of the advantages of the CFS is that it incorporates components of three geriatric domains: the somatic domain (comorbidity), the functional domain (functional level) and the mental domain (cognition) [34]. Patients with scores of 5 or higher are at an increased risk for frailty and require further evaluation of the frailty status [34]. The CFS and the G8 (Table 1) are increasingly adopted as they have been consistently predictive of adverse outcomes and mortality in different settings [34,35].

Indirect frailty screening methods use clinical data from Electronic Health Records (EHR), also called administrative frailty tools [37]. The methodology of these assessment methods is often based on the model of ‘’accumulation of deficits’’, as previously described [38]. An advantage of these administrative assessment methods is that they allow frailty screening in large cohorts of patients.

The majority of the included studies used an administrative frailty assessment method (Table 2). Six studies used the Hospital Frailty Risk Score (HFRS) [39,40,41,42,43,44]. The HFRS is a frailty screening method that is based on the International Statistical Classification of Diseases and Health-Related Problems, tenth revision (ICD-10) coding systems [45]. This is a computerized method that generates a score based on the type and number of ICD-10 diagnoses generated from the medical record of a patient [45]. One other study used a similar administrative assessment method: the Johns Hopkins ACG frailty-defining diagnoses indicator [46]. Three studies used a comorbidity-based frailty assessment method using ICD data abstracted from medical records. One study used the Modified Frailty Index (mFI) [47], and one used a simplified version [48]. The Simplified Frailty Index (sFI) contains five selected items of the twelve in the mFI [48]. Another comorbidity-based tool is the ‘’frailty trait count’’, which consists of the five items in the sFI with one additional item [49]. Two studies measured frailty using a geriatric assessment in patients with IBD aged 65 years and older [50,51]. They conducted a geriatric assessment that explored five geriatric domains: the somatic domain (multimorbidity, malnutrition, polypharmacy), activities of daily living (ADL, IADL), physical capacity (handgrip strength, gait speed), the mental domain (depression, cognitive function) and the social domain (presence of a life partner) [50].

### 3.3. What Is the Prevalence of Frailty in Patients with IBD?

We identified twelve studies that examined frailty in patients with IBD. Details about the included studies are shown in Table 2. The prevalence of (high risk of) frailty appears to be higher in patients with IBD compared to a matched non-IBD control population (6% vs. 12%, respectively) [42].

The presence of frailty in the study by Asscher et al. [50] was defined as deficits in two or more geriatric domains and was established in 47.4% of older patients with IBD. This study has provided insight in which geriatric domains are most often affected in older patients with IBD. The somatic domain was most often affected (51.6%), followed by impaired activities of daily living (43.0%), the social domain (23.7%), physical activity (22.7%) and lastly the mental domain (16.5%) (Table 3). Another paper by Asscher et al. [51] reported the use of a frailty screening tool, the Geriatric-8 (G8). This tool classified 48% of patients with IBD 65 years or older at risk of being frail [51].

In the studies that used an administrative frailty assessment method, the prevalence of frailty varied between 6% and 39.3%. An important caveat is that in some of these studies, no age threshold was applied. This is reflected in the mean age of the participants (Table 2), and will have led to a lower prevalence of frailty in these studies. Prevalence of frailty in surgical patients was slightly higher, probably reflecting a patient population with a higher IBD disease burden. However, again no age thresholds were used in these studies. Although frailty is not limited to a certain age threshold and can occasionally be found in younger patients, the multidimensional decline that contributes to development of frailty is ageing-related, increasing the prevalence of frailty with ageing. Therefore, these studies do not tell us about the prevalence in older patients with IBD.

### 3.4. What Factors Increase the Risk for the Onset or Progression of Frailty in Community Dwelling Older Adults?

Risk factors for frailty are patient characteristics or conditions that are known to attribute to the multidimensional process of physical decline, eventually leading to the condition frailty. The number and type of risk factors can vary depending on the characteristics of the investigated population. Some risk factors consistently associate with an increased risk of frailty in different settings, while other risk factors are unique for a specific condition or population. For example, the presence of disease activity in chronic inflammatory diseases such as rheumatoid arthritis or IBD has been associated with increased risk of frailty [50,52].

Previous studies identified risk factors for the onset or progression of frailty in community-dwelling older adults [12,53]. They reported a broad range of risk factors categorized in sociodemographic factors, clinical factors, lifestyle factors and biological factors. Alternatively, these risk factors can be stratified based on the geriatric domain they affect (Table 4), enabling easier identification of geriatric domains at risk of frailty.

### 3.5. What Factors Increase the Risk for the Onset or Progression Frailty in Patients with IBD?

Three papers have examined risk factors that are associated with frailty in patients with IBD [43,44,50]. Increasing age was reported as a risk factor for frailty in patients with IBD [43,50]. The role of female sex as a risk factor for frailty in patients is less clear: one study demonstrated a strong association between female sex and risk for geriatric deficits (adjusted Odds Ratio (aOR) 1.94, [95% confidence interval (CI) 1.26–2.98], *p*-value 0.002) [50], whereas another study did not (aOR 1.17, [95% CI 0.99–1.38], *p*-value 0.060) [43].

Older patients with IBD are frequently affected by multimorbidity and polypharmacy [54,55]. Kochar et al. [43] found that the presence of ≥1 comorbidity was the strongest predictor of frailty in a multivariable analysis, as it was associated with an 17.23 odds of frailty ([95% CI 8.11–36.63], *p*-value < 0.001).

### 3.6. Which IBD-Specific Factors Are Associated with an Increased Risk of Frailty?

CD and UC have distinct disease behavior and characteristics and some disease characteristics may contribute to the development of frailty. Two studies found that the presence of CD (compared to UC) was associated with an increased odds of frailty (aOR 1.35, [95% CI 1.14–1.61], *p*-value < 0.001) (aOR 1.80 [95% CI 1.18–2.74], *p*-value 0.006) [43,50].

**Figure 2:**
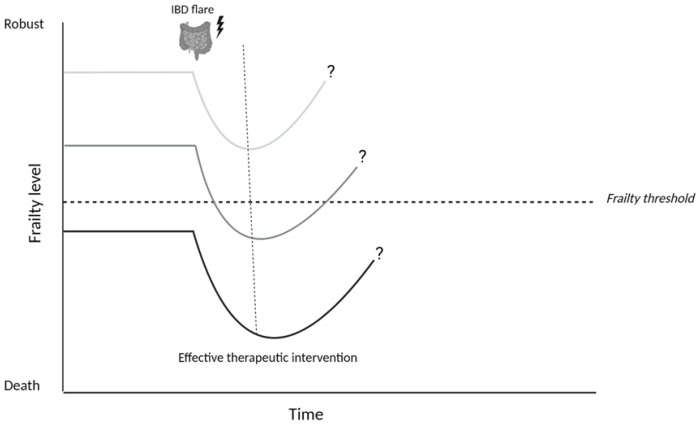
Hypothesized effect of an IBD flare on frailty in patients with Inflammatory Bowel Disease. An IBD flare should be considered as a potential stressor that can cause a temporary or definite decline in the degree of frailty (level). The impact of this stressor depends on the frailty level prior to the IBD flare, which consists of existing risk factors for frailty in a patient. Achieving disease remission and thereby eliminating the stressor, enables (partial) recovery of frailty levels to baseline. It is likely that this recovery phase takes longer in patients who were already frail prior to the IBD-flare, because they already had a reduced reserve capacity. Images were created using biorender.com.

Disease activity was found as an important factor in relation to frailty. Asscher et al. [50] reported an independent association between disease activity and the presence of deficits in the geriatric assessment, this was seen for both biochemical (aOR 3.36, [95% CI 1.94–5.83], *p*-value 0.000) and clinical disease activity (aOR 2.19, [95% CI 1.28–3.74], *p*-value 0.004). Disease activity was the strongest associated factor in relation to frailty in this study. Conversely, treating disease activity in frail, older patients with IBD was associated with reduced post-treatment frailty [44], especially in those patients with a higher baseline level of frailty. Inflammatory activity can introduce potential risk factors for frailty into different geriatric domains. For example, inflammation can induce loss of skeletal muscle and may eventually lead to sarcopenia [27]. In addition, disease activity is described as a risk factor for malnutrition and polypharmacy [56,57]. Active disease can also impact the mental domain as both mood disorders and cognitive performance have been associated with disease activity over time in IBD [58,59]. These data reflect the important role of disease activity contributing to frailty in patients with IBD and the dynamic character of frailty: the degree of frailty can vary over time depending on the presence or absence of contributing risk factors (Figure 2).

Therapeutic strategies for the management of patients with IBD often consist of corticosteroids, immunomodulators or biologicals [3]. Older patients are susceptible to developing a range of potential adverse outcomes, especially related to long-term use of corticosteroids (e.g., diabetes, sarcopenia, glucocorticoid-induced osteoporosis) [60,61]. These adverse outcomes could function as risk factors to the onset or progression of frailty. In the IBD literature, one study reported an association between corticosteroid use at baseline and risk of frailty (aOR 1.45, [95% CI 1.21–1.75], *p*-value < 0.001) [43], whereas another study did not find this association [50]. No association was found between the use of immunomodulators or biologicals and an increased risk of frailty [43,50].

Kochar et al. [43] reported both previous IBD-related hospitalization and previous IBD-related surgery to be associated with an increased risk of frailty, whereas Asscher et al. [50] did not. Both studies found previous all-cause hospitalization as a risk factor for frailty in patients with IBD [43,50]. The association between frailty and hospitalization appears bidirectional, as frailty is established as a predictor for adverse outcomes such as mortality and hospitalizations, and previous hospitalizations also associate with risk of frailty. During a hospital admission, frailty is associated with functional decline, increased vulnerability to complications and other adverse health outcomes [62,63].

### 3.7. What Is the Prognostic Value of Frailty in Patients with IBD?

**Figure 3:**
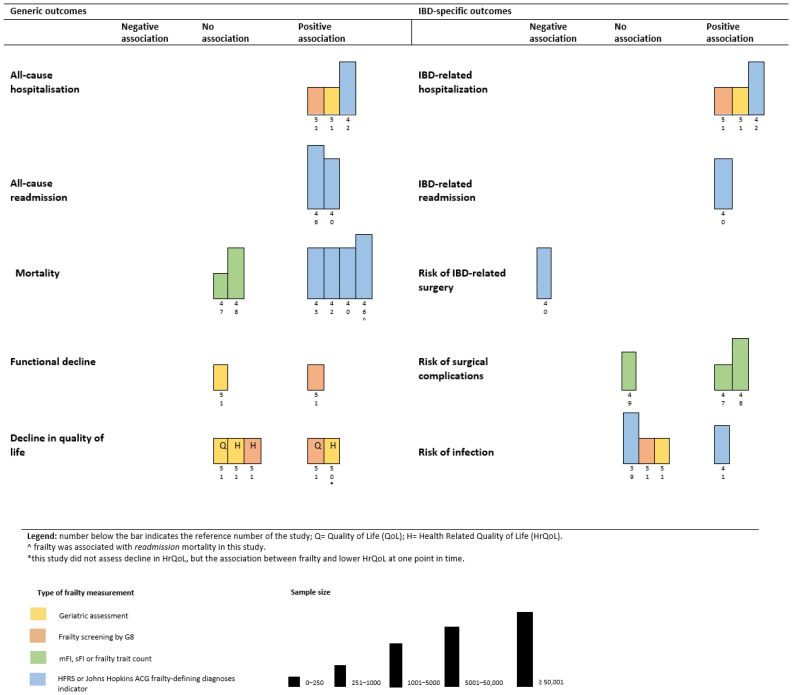
Harvest plot of the association between frailty and (1) generic outcomes and (2) IBD-specific outcomes.

In this section, we will review the impact of frailty on generic outcomes and IBD-specific outcomes (Figure 3). Frailty was consistently associated with an increased risk for all-cause hospitalization and all-cause re-admission. Kochar et al. [42] reported a HR 2.42 ([95% CI 2.24–2.61]) for all-cause hospitalization in their cohort. In addition, Asscher et al. [51] reported a positive association between severe geriatric deficits and increased risk for all-cause hospitalizations. In addition, deficits in geriatric domains were associated with acute and IBD-related hospitalizations [51]. They also examined the association between an increased risk of frailty at baseline and the occurrence of all-cause and acute hospitalizations at follow-up. Risk of frailty was associated with acute hospitalizations (aHR 2.21, [95% CI 1.27–3.87], *p*-value 0.005), but not with all-cause hospitalizations (aHR 1.53, [95% CI 0.96–2.44], *p*-value 0.074) [51]. Frailty was associated with an increased risk for all-cause readmission in the papers of Faye et al. [46] (adjusted risk ratio (aRR) 1.16, [95% CI 1.14–1.17], *p*-value < 0.01) and Qian et al. [40] (aHR 1.21, [95% CI 1.17–1.25], *p*-value < 0.01).

Findings from studies on the association between frailty and mortality differed depending on the setting and the frailty tool used. Two studies in a surgical IBD-setting that used a comorbidity-based frailty tool found no association between frailty and mortality [47,48]. Four studies that used the HFRS, all reported a strong association between frailty and mortality ((aOR 2.90 [95% CI 2.29–3.68); (HR of 3.22 [95% CI 2.86–3.61]); (aHR 1.57 [95% CI 1.34–1.83], *p*-value < 0.01); (aRR 1.12 ([95% CI 1.02–1.23], *p*-value 0.02)) [40,42,43,46]. The role of frailty in the surgical IBD setting as a predictor for mortality was not demonstrated, yet there is consistent evidence that frailty is associated with an increased risk for mortality in non-surgical IBD setting when the HFRS is applied.

Functional decline and decreased quality of life were evaluated as outcomes of frailty by Asscher et al. [50,51]. Frailty, reflected by the number of geriatric deficits, was associated with lower health-related quality of life (HRQoL) [50]. In their paper with follow-up data, they demonstrated that risk of frailty at baseline was associated with a decline in the quality of life (QoL) (aOR 2.14 [95% CI 1.26–3.62], *p*-value 0.005) and functional status (IADL) (aOR 3.64 [95% CI 1.65–8.00], *p*-value 0.001) after 18 months [51]. Interestingly, frailty measured by a geriatric assessment at baseline was not associated with a decline in QoL or functional status at follow-up [50].

The results on the association between frailty and an increased risk of infections in IBD were inconclusive. Two studies reported no association between frailty or risk of frailty and increased risk of infections [39,51], whereas one study found frailty associated with an increased risk of infections in both anti-tumor necrosis factor and immunomodulator therapy [41]. Three studies assessed the association between frailty and risk for morbidity following surgery. Telemi et al. [47] (aOR 25.5; *p*-value ≤ 0.001) and Wolf et al. [48] (aOR 2.59 [95% CI 1.84–3.63], *p*-value < 0.0001) reported a positive association between frailty and overall morbidity following surgery. However, Cohan et al. [49] did not find this association.

## 4. Conclusions and Future Directions

In this scoping review we aimed to summarize the current literature on frailty in IBD. We aimed to describe (1) frailty assessment methods, (2) the prevalence of frailty in IBD, (3) risk factors for frailty in IBD and (4) the prognostic value of frailty in IBD. The majority of the studies that were included in this review used an administrative frailty assessment method, most commonly the HFRS. Only two studies used a geriatric assessment to measure frailty. Overall, the prevalence of frailty greatly varied and depended on the population and frailty assessment method. In addition, as expected, frailty was more prevalent in older patients. The risk factors for frailty that were found in patients with IBD include increasing age, presence of comorbidity, Crohn’s disease (CD), clinical and biochemical disease activity and previous all-cause hospitalization. Frailty was linked to a range of adverse outcomes that include an increased risk for all-cause hospitalization, all-cause readmission, mortality in non-surgical setting and IBD-related hospitalization and readmission.

Implementing frailty into clinical care for patients with IBD does not have to be complicated or time-consuming. Identification of those patients at risk of frailty is the most important aspect, as this allows for further assessment and intervention. There is growing evidence that frailty is most amendable to intervention in the early stages, urging early identification in the beginning of the disease process of frailty [64]. Therefore, periodic evaluation of frailty status should be part of clinical care, especially in older patients with IBD, because frailty is a dynamic condition that can fluctuate depending on the presence of risk factors and stressors. We propose the Clinical Frailty Scale (CFS) as the frailty screening method in all patients with IBD, although this scale is not yet validated in this population. The CFS has shown excellent performance in a wide variety of patient populations and is recommended as frailty screening instrument by the International Conference of Frailty and Sarcopenia Research (ICFSR) [34,64].

There is increasing evidence that physical frailty can be prevented or reversed by the application of combined nutritional and physical exercise intervention programs [65]. To date, no studies have been conducted on the impact of these interventional programs on frailty in patients with IBD. However, in other medical fields the efficacy of interventional programs has already been demonstrated [66]. Interventional programs might not only improve physical frailty, but also positively impact on therapy outcomes [67]. For example, a randomized controlled trial investigated the impact of CGA-based interventions (medication changes, nutritional therapy and physiotherapy) in frail patients receiving adjuvant chemotherapy for colorectal carcinoma [67]. More patients in the intervention group completed planned chemotherapy without further dose reductions or delay compared to patients receiving standard of care [67].

The effective management of patients with increased risk of frailty or frailty also includes incorporating frailty in clinical decision making and therapeutic management strategies. The use of frailty as a risk construct instead of age and comorbidity allows for the better selection of patients who are ‘fit’ for a certain surgical or pharmacological treatment. To date, evidence-based guidelines for treatment of older patients with IBD are lacking and older patients are often excluded from clinical trials, as recently stressed by Vieujean et al. [68]. Frailty could be integrated into treatment selection to tailor pharmaceutical management, for example, by adjusting therapy regimens or reducing dose therapy in patients with frailty. A positive effect of these ‘’tailored’’ approaches in patients with frailty were demonstrated in oncology with adjusted chemotherapy regimens [69].

Future research on frailty in IBD should focus on the identification of the optimal frailty screening method to identify patients with IBD at increased risk of frailty. We demonstrated that frailty outcomes varied depending on the frailty assessment method and the age of the investigated population. Different frailty risk factors and outcomes may apply depending on age and setting. Reanalyzing previous data, stratified by patients’ age specifically, might provide more insight into the frailty syndrome in younger and older patients. Moreover, tailored treatment strategies, such as adjusted therapy regimens or multicomponent intervention programs for patients with frailty in IBD are imaginable, however, research is needed on this topic.

## Figures and Tables

**Table 1:** An overview of two commonly used frailty screening methods: the Clinical Frailty Scale and the Geriatric-8.

Clinical Frailty Scale [10]		Geriatric-8 [36]	
1. Very Fit	People who are robust, active, energetic and motivated. These people commonly exercise regularly. They are among the fittest for their age.	1. Has food intake declined over the past 3 months due to loss of appetite, digestive problems, chewing or swallowing difficulties?	0: severe decrease in food intake
			1: moderate decrease in food intake
			2: no decrease in food intake
2. Well	People who have no active disease symptoms but are less fit than category 1. Often, they exercise or are very active occasionally, e.g., seasonally.	2. Weight loss during the last 3 months	0: weight loss > 3 kg
			1: does not know
			2: weight loss between 1 and 3 kgs
			3: no weight loss
3. Managing Well	People whose medical problems are well controlled, but are not regularly active beyond routine walking.	3. Mobility	0: bed or chair bound
			1: able to get out of bed/chair but does not go out
			2: goes out
4. Vulnerable	While not dependent on others for daily help, often symptoms limit activities. A common complaint is being “slowed up” and/or being tired during the day.	4. Neuropsychological problems	0: severe dementia or depression
			1: mild dementia or depression
			2: no psychological problems
5. Mildly Frail	These people often have more evident slowing, and need help in high order IADLs (finances, transportation, heavy housework, medications). Typically, mild frailty progressively impairs shopping and walking outside alone, meal preparation and housework.	5. Body Mass Index	0: BMI < 19
			1: BMI 19 to <21
			2: BMI 21 to <23
			3: BMI 23 or greater
6. Moderately Frail	People need help with all outside activities and with keeping house. Inside, they often have problems with stairs and need help with bathing and might need minimal assistance (cuing, standby) with dressing.	6. Takes more than 3 medications per day?	0: yes 1: no
7. Severely Frail	Completely dependent for personal care, from whatever cause (physical or cognitive). Even so, they seem stable and not at high risk of dying (within ~6 months).	7. In comparison with other people of the same age, how does the patient consider his/her health status?	0: not as good
			0.5: does not know
			1: as good
			2: better
8. Very Severely Frail	Completely dependent, approaching the end of life. Typically, they could not recover even from a minor illness.	8. Age	0: >85
			1: 80–85
			2: <80

9. Terminally Ill	Approaching the end of life. This category applies to people with a life expectancy < 6 months, who are not otherwise evidently frail.		

**Table 2:** Study characteristics, frailty measurement methods and frailty prevalence.

Author and Year of Publication	Study Sample	Age			Population	Frailty Assessment Method	Data Source	Frailty Prevalence
Asscher et al. [50] (2022)	*n* = 405	70 years (67–74)			Patients ≥ 65 years or older Outpatient setting IBD	Geriatric assessment	Prospective cohort study	47.4% (moderate to severe geriatric deficits in geriatric assessment)
Asscher et al. [51] (submitted manuscript)	*n* = 405	70 years (67–74)			Patients ≥ 65 years or older Outpatient setting IBD	Geriatric assessment Geriatric frailty screening	Prospective cohort study	47.4% (moderate to severe geriatric deficits in geriatric assessment) 48% (at risk of frailty)
Telemi et al. [47] (2018)	*n* = 943	46 years (33–59)			No age criteria. Patients undergoing colectomy UC	Modified Frailty Index (mFI)	National Surgical Quality Improvement Program database	32.3% (mFI score > 0)
Wolf et al. [48] (2021)	*n* = 9.023	sFI = 0: 18–64 years = 94%, 65–79 years = 4.9%, >80 years = 0.3% sFI = 1: 18–64 years = 69.5%, 65–79 years = 24.9%, >80 years = 2.7% sFI ≥ 2: 18–64 years = 54.2%, 65–79 years = 38.2%, >80 years = 3.8%			No age criteria. Patients undergoing bowel resection. CD	Simplified Frailty Index (sFI)	National Surgical Quality Improvement Program database	17.8 % (sFI score > 0)
Cohan et al. [49] (2015)	*n*= 2.493	≤50 years: 34 years (range 18–50) >60 years: 64.5 years (range 61–90)			No age criteria. Patients undergoing total proctocolectomy with ileal pouch-anal anastomosis or completion proctocolectomy with IPAA UC	Frailty trait count	National Surgical Improvement Program database	53.9% 1 or more frailty trait counts (in patients > 60 years old)
Faye et al. [46] (2021)	*n* = 1.405.529	Age (years)	Frail (*n*, %)	Not frail (*n*, %)	No age criteria. Patients admitted to the hospital IBD	Johns Hopkins Adjusted Clinical Groups	Nationwide Readmission Database	10.9%
		<18	8.974 (13.83%)	55.926 (86.17%)				
		18–30	21.919 (10.00%)	197.055 (90.00%)				
		31–40	16.602 (8.00%)	190.783 (82.00%)				
		41–50	19.223 (9.11%)	191.787 (90.89%)				
		51–65	35.397 (10.52%)	300.947 (89.48%)				
		66–80	33.415 (12.81%)	227.507 (87.19%)				
		>80	17.443 (16.46%)	88.549 (83.54%)				
Kochar et al. [41] (2020)	Anti-TNF cohort *n* = 1.299 Immunomodulator cohort *n* = 2.676	*Anti-TNF cohort* Fit: 35 years (25–50) Frail: 41 years (28–53) *Immunomodulator cohort* Fit: 38 years (26–53) Frail: 44 years (32–61)			No age criteria. Patients receiving immunosuppressive therapy IBD	Hospital Frailty Risk Score (HFRS) derived frailty-related diagnosis code	Electronic Health Record	8% in patients > 60 years treated with anti-TNF agent 12% in patients > 60 years treated with immunomodulator
Kochar et al. [42] (2022)	*n* = 10.590 (IBD) *n* = 103.398 (matched comparators)	71 years ± 8 years			Patients aged ≥ 60 years Cohort IBD	Hospital Frailty Risk Score	Electronic Health record	12% (higher risk of frailty)
Kochar et al. [43] (2020)	*n* = 11.001	Fit: 46 years (32–61) Frail: 53 years (40–69)			No age criteria. Cohort IBD	Hospital Frailty Risk Score derived frailty-related diagnosis code	Electronic Health Record	6%
Qian et al. [40] (2021)	*n* = 47.402	Non-frail: 49.2 years ± 18.5 Frail: 61.9 years ± 18.2			Adults ≥ 18 years Admitted with a primary or secondary diagnosis of IBD IBD	Hospital Frailty Risk Score	Nationwide Readmissions database	32.7%
Singh et al. [39] (2021)	*n* = 5.987	Not frail: 40 years ± 14 Frail: 44 years ± 17			Adult patients (18–89 years) Patients treated with a biological IBD	Hospital Frailty Risk Score	OptumLabs Data Warehouse database	39.3%
Kochar et al. [44] (2021)	*n* = 1.210	Not frail: 33.9 years ± 15.8 Frail: 36.9 years ± 17.1			No age criteria. Patients initiating anti-TNF agents IBD	Hospital Frailty Risk Score	Electronic Health Record	15.6%

Abbreviations: CD, Crohn’s disease; IBD, inflammatory bowel disease; UC, ulcerative colitis; TNF, tumor necrosis factor.

**Table 3:** Prevalence and type of frailty defining diagnosis per study.

Geriatric Assessment					
Asscher et al. [50,51]	Impaired somatic domain (51.6%) Comorbidity (13.8%) Polypharmacy (40.2%) At risk of malnutrition (18.1%) Malnutrition (2.0%)	Impaired in activities of daily living (43.0%) Impaired in ADL (29.9%) Impaired in ADL (23.2%)	Impaired in social domain (23.7%) No life partner (23.7%)	Impaired in physical capacity (22.7%) Low handgrip strength (19.9%) Low gait speed (6.0%)	Impaired in mental domain (16.5%) Cognitive impairment (10.1%) Depressive symptoms (8.7%)
**Electronic health record base**					
Telemi et al. [47]	NR				
Wolf et al. [48]	Hypertension (15.8%)	Diabetes (3.3%)	Chronic Obstructive Pulmonary Disease (1.5%)	Dependent functional status (0.7%)	Congestive heart failure (0.1%)
Cohan et al. [49] *	Hypertension (46.5%)	Diabetes (13.4%)	Preoperative weight loss (5.1%)	Chronic obstructive pulmonary disease (2.4%)	Functional dependence (0.8%)
Faye et al. [46]	Malnutrition (55%)	Weight loss (20%)	Presence of a decubitus ulcer (11%)		
Kochar et al. [41]	NR				
Kochar et al. [42] **	Comorbidity-related diagnosis: Non-infective gastroenteritis and colitis (41.45%) Other functional intestinal disorders (13.52%) Other diseases of digestive system (13.41%)	Function-related diagnosis: Non-traumatic compartment syndrome (8.66%) Fall on same level from slipping, tripping and stumbling (8.02%) Fall (5.16%)	Cognition-related diagnosis: Sequelae of cerebrovascular disease (4.37%) Depressive episode (3.02%) Other symptoms and signs involving cognitive function and awareness (1.37%)	Sensory-related diagnosis: Hearing loss (1.01%) Speech disturbances (0.76%) Blindness and low vision (0.50%)	
Kochar et al. [43]	Protein energy malnutrition (74%)	Walking difficulty (20%)	Unspecified protein malnutrition (8%)		
Singh et al. [39]	Hypokalemia (9.4%)	Urinary tract infection (8.2%)	Constipation (7.8%)	Dehydration (7.3%)	Joint pain (4.7%)
Qian et al. [40]	Disorders of fluid electrolyte and acid-base balance (47.8%)	Other and unspecified anemias (24.7%)	Personal history of certain diseases (13.4%)	Acute renal failure (11.5%)	Chronic kidney disease (9.4%)
Kochar et al. [44]	NR				

Abbreviations: NR, not reported. * Data presented of age group > 60 years or older. ** The original article contains a supplementary table with the prevalence of all ICD codes associated with frailty in this population. The three most prevalent diagnoses per diagnosis category are presented here.

**Table 4:** Risk factors for frailty in community-dwelling older adults categorized by geriatric domain.

Geriatric Domain	Risk Factor for Frailty
Increasing age and female sex	
Somatic domain	Chronic diseases Polypharmacy Obesity Underweight Malnutrition Lifestyle factors: smoking and increased alcohol intake Micronutrients deficiency (low carotenoids, vitamin B6, vitamin D and vitamin E) Endocrine factors (androgen deficiency and IGF-1)
Mental domain	Impaired cognition Depressive symptoms
Physical capacity	Physical inactivity
Social domain	Lower educational level Ethnic minority Low socioeconomic position Patients living alone or experiencing loneliness

## Data Availability

Not applicable.
